# Regulation of differentiation- and proliferation-inducers on Lewis antigens, **α**-fucosyltransferase and metastaticotential in hepatocarcinoma cells

**DOI:** 10.1054/bjoc.2001.1815

**Published:** 2001-06

**Authors:** F Liu, H-L Qi, H-L Chen

**Affiliations:** Key Laboratory of Glycoconjugate Research, Ministry of Health, Department of Biochemistry, School of Medicine, Fu-Dan University, Shanghai, 200032, China

**Keywords:** human hepatocarcinoma cells, differentiation-inducer, proliferation-inducer, Lewis antigen, α-fucosyltransferase, metastasis-related phenotype

## Abstract

The expressions of Lewis (Le) antigens, α-1,3/1,4 fucosyltransferases (α-1,3/1,4 FuTs), and metastatic potential after the treatment of 2 differentiation inducers, all- *trans* retinoic acid (ATRA), 8-bromo-cyclic 3′,5′adenosine monophosphate (8-Br-cAMP); and 2 proliferation inducers, epidermal growth factor (EGF) and phobol-12-myristate-13-acetate (PMA), on 7721 human hepatocarcinoma cell line were studied. Cell adhesion to human umbilical vein endothelial cells (HUVEC), cell migration through transwell and invasion through matrigel were selected as the indexes of metastatic potential-related phenotypes. Using fluorescence-labelled antibodies and flow-cytometric analysis, it was found that 7721 cells mainly expressed sialyl Lewis X (SLe^x^) and a less amount of sialyl dimeric Lewis X (SDLe^x^) antigens on the cell surface. Their expressions were down-regulated by ATRA, and up-regulated by EGF. SLe^x^antigen was also decreased and increased by the treatment of 8-Br-cAMP and PMA respectively. With Northern blot to detect the mRNAs of α-1,3/1,4 FuTs, the main enzymatic basis for the change in SLe^x^expression was found to be the alteration of the expression of α-1,3 FuT-VII. It was evidenced by the observations that α-1,3 FuT-VII was the main α-1,3/1,4 FuT in 7721 cells, while α-1,3/1,4 FuT-III and α-1,3 FuT-VI were expressed rather low. The changes in the expressions of SLe^x^antigen and α-1,3 FuT-VII resulted in the altered cell adhesion to tumour necrosis factor-α stimulated HUVEC, since only the monoclonal antibody of the SLe^x^, but not other monoclonal antibodies blocked the adhesion of 7721 cells to HUVEC. The migration and invasion of 7721 cells were also reduced by the treatment of ATRA or 8-Br-cAMP, and elevated by EGF or PMA. The above findings indicate that the metastatic potential of 7721 cells is suppressed by differentiation-inducers and promoted by proliferation-inducers. © 2001 Cancer Research Campaign http://www.bjcancer.com


*British Journal of Cancer* (2001) **84**(11), 1556–1563
© 2001 Cancer Research Campaign
doi: 10.1054/ bjoc.2001.1815, available online at http://www.idealibrary.com on IDEAL®

http://www.bjcancer.com

### Regulation of differentiation- and proliferation-inducers on Lewis antigens, α-fucosyltransferase and metastatic potential in hepatocarcinoma cells

**F Liu*, H-L Qi* and H-L Chen**

Key Laboratory of Glycoconjugate Research, Ministry of Health, Department of Biochemistry, School of Medicine, Fu-Dan University, Shanghai, 200032, China

**Summary** The expressions of Lewis (Le) antigens, α-1,3/1,4 fucosyltransferases (α-1,3/1,4 FuTs), and metastatic potential after the treatment of 2 differentiation inducers, all-*trans* retinoic acid (ATRA), 8-bromo-cyclic 3′,5′adenosine monophosphate (8-Br-cAMP); and 2 proliferation inducers, epidermal growth factor (EGF) and phobol-12-myristate-13-acetate (PMA), on 7721 human hepatocarcinoma cell line were studied. Cell adhesion to human umbilical vein endothelial cells (HUVEC), cell migration through transwell and invasion through matrigel were selected as the indexes of metastatic potential-related phenotypes. Using fluorescence-labelled antibodies and flow-cytometric analysis, it was found that 7721 cells mainly expressed sialyl Lewis X ($SLe^x$) and a less amount of sialyl dimeric Lewis X ($SDLe^x$) antigens on the cell surface. Their expressions were down-regulated by ATRA, and up-regulated by EGF. $SLe^x$ antigen was also decreased and increased by the treatment of 8-Br-cAMP and PMA respectively. With Northern blot to detect the mRNAs of α-1,3/1,4 FuTs, the main enzymatic basis for the change in $SLe^x$ expression was found to be the alteration of the expression of α-1,3 FuT-VII. It was evidenced by the observations that α-1,3 FuT-VII was the main α-1,3/1,4 FuT in 7721 cells, while α-1,3/1,4 FuT-III and α-1,3 FuT-VI were expressed rather low. The changes in the expressions of $SLe^x$ antigen and α-1,3 FuT-VII resulted in the altered cell adhesion to tumour necrosis factor-α stimulated HUVEC, since only the monoclonal antibody of the $SLe^x$, but not other monoclonal antibodies blocked the adhesion of 7721 cells to HUVEC. The migration and invasion of 7721 cells were also reduced by the treatment of ATRA or 8-Br-cAMP, and elevated by EGF or PMA. The above findings indicate that the metastatic potential of 7721 cells is suppressed by differentiation-inducers and promoted by proliferation-inducers. © 2001 Cancer Research Campaign http://www.bjcancer.com

**Keywords**: human hepatocarcinoma cells; differentiation-inducer; proliferation-inducer; Lewis antigen; α-fucosyltransferase; metastasis-related phenotype

Liver carcinoma is one of the important cancers in China with high incidence. It was reported by our laboratory that a human hepatocarcinoma cell line, 7721, was shown to be differentiated by all-*trans* retinoic acid (ATRA) and the cell permeable derivatives of cyclic 3′,5′-adenosine monophosphate (cAMP). After treatment with ATRA or cAMP derivatives, the morphological appearance was changed toward normal (Ai et al, 1990, 1991a), the activities of γ-glutamyltransferase (GGT) and tyrosine protein kinase (TPK) (Chai et al, 1993a), protein kinase C (PKC) (Chai and Chen, 1994a), as well as the expressions of α-fetal protein (Ai et al, 1990) and some oncogenes (Ai et al, 1991b; Chai and Chen, 1994b) were decreased, while the expression of albumin was up-regulated by ATRA (Ai et al, 1990). On the contrast, cell growth, the activities of TPK and GGT (Chai et al, 1993b; Shen et al, 1993; Xia et al, 1998), as well as the expressions of some oncogenes (Chai et al, 1994c), were increased during the treatment of phorbol-12-myristate-13-acetate (PMA) or epidermal growth factor (EGF). Therefore, ATRA and cAMP derivatives can be considered as the differentiation-inducers of 7721 cells, while PMA and EGF are the proliferation-inducers of this cell line. On the other hand, we found that a metastasis-related glycan processing enzyme, N-acetylglucosaminyltransferase V (GnT-V) (Taniguchi et al, 1999), and its product, GlcNAcβ1,6Manα-branching structure in asparagine-linked glycans, were also down-regulated by ATRA or dibutyl-cAMP (Dong et al, 1994; Chen et al, 1995) and up-regulated by EGF or PMA (Xia et al, 1998).

It was well-documented that Lewis antigens, mainly located on the outer chains of the glycans of cell surface glycolipids or the O-linked glycans of glycoproteins, were closely related to the metastatic potential and overall prognosis of many human cancers, such as colorectal, prostate and lung cancer (Nakamori et al, 1993; Ogawa et al, 1994; Jorgensen et al, 1995). Lewis antigens are a series of fucosylated Galβ1,3/1,4GlcNAc containing oligosaccharides (sialylated or not sialylated, β1,3 linkage in type 1 chain and β1,4 linkage in type II chain), and are known to participate in the process of leukocyte infiltration during inflammation and cell adhesion during malignant cell metastasis. In the beginning of haematogeneous metastasis, malignant cells have to first invade into blood vessels. After their dissemination via the circulation, they may adhere to and penetrate through the vascular endothelium, and move into the surrounding tissue to form metastatic colonies (Hakomori, 1996). The E-or P-selectin expressed on the surface of vascular endothelial cells interacts with sialyl Lewis antigens, such as $SLe^x$, $SDLe^x$ and $SLe^a$, expressed on the surface

*Received 11 September 2000*
*Revised 1 February 2001*
*Accepted 9 February 2001*

*Correspondence to:* H-L Chen

*These two authors contributed equally to this manuscript.

of malignant cells, and mediates the adhesion of malignant cells to the vascular endothelium (Takada et al, 1993; Kanas et al, 1996). Sialyl Lewis antigen, therefore, are considered as another metastasis-related structure of glucans in addition to GlcNAcβ1, 6Manα-branch.

A set of glycosyltransferases is responsible for the biosynthesis of Lewis antigens. The last step in their synthesis, fucosylation, is catalysed by α1,3/1,4 fucosyltransferases (α1,3/1,4 FuTs). 6 human α1,3/1,4 FuTs have been cloned (αFuT-III, IV, V, VI, VII and IX) (Kukowska-latallo et al, 1990; Lowe et al, 1991; Weston et al, 1992a, 1992b; Sasaki et al, 1994; Kudo K et al, 1998). 4 of them efficiently fucosylate sialylated acceptors, while α1,3 FuT-IV and α1,3 FuT-IX prefer non-sialylated neutral acceptors. α1,3/1,4FuT-III is the only αFuT, which has 2 different activities (α1,3 and α1,4 fucosylation), leading to the generation of $SLe^x$ and $SLe^a$, respectively. However, α1,3 FuT-VII only catalyses the synthesis of sialylated $Le^x$ ($SLe^x$) (Sasaki et al, 1994; Narimatsu, 1998). There were many literatures which reported that the expressions of α1,3/1,4 FuTs are positively related to the metastasis potency of some cancers, and negatively related to the prognosis of the patients (Ito et al, 1997; Ogawa et al, 1997; Kudo T et al, 1998).

On the base of our previous studies, the effects of 2 differentiation inducers, ATRA and 8-Br-cAMP, as well as 2 proliferation inducers, PMA and EGF, on the expressions of Lewis antigens on 7721 cell surface, their enzymatic mechanisms, and their relations to some metastatic phenotypes ex vivo were investigated.

### MATERIALS AND METHODS

The 7721 human hepatocarcinoma cell line was obtained from the Institute of Cell Biology, Academic Sinica. Plasmids containing cDNAs of α1,3 FuTs, monoclonal antibodies (mAb) of $SLe^x$ and $SLe^a$, KM 93 and CA19-9 respectively, were kindly gifted from Prof Narimatsu at Soka University. FH6 (mAb of $SDLe^x$) was a gift from Dr Hakomori at University of Washington. CD15 (mAb of $Le^x$) was purchased from Dako. The cDNA of glyceraldehyde-3-phosphate dehydrogenase (GAPDH) was from Dr Shimizu at Tokushima University. Human umbilical vein endothelial cells (HUVEC) were obtained from the Department of Anatomy of our university. RPMI 1640 medium, DMEM medium and Matrigel were from GIBCO. ATRA, 8-Br-cAMP, PMA, EGF, L-poly-L-lysine, Fluorescein isothiocynate (FITC)-conjugated goat antibodies against mouse IgM or IgG, and sialidase (*Clostridium perfringens*) were purchased from Sigma. Trizol, DNA restriction endonucleases and random primer labelling kit were from Promega. Hybond-N+ nylon membrane and (α-$^{32}$P)-dATP were from Amersham Corp. Insert (transwell) and cell culture plates were obtained from NUNC Company. Other reagents were commercially available in China.

### Cell culture and treatment

Cells were cultured at 37˚C, 5% $CO_2$ in RPMI-1640 medium containing 10% FCS, penicillin and streptomycin as previously described by our laboratory (Ai et al, 1990). ATRA, PMA in ethanol or 8-Br-cAMP, EGF in re-distilled water were separately added to the culture medium, and cultured for 48 h. The final concentrations of these compounds in the medium were indicated in the text, tables or the legends of figures. The culture medium of the control cells for ATRA or PMA contained the same final concentration of ethanol, but all the results were no different between the ethanol-containing medium and the ethanol-free medium for EGF and 8-Br-cAMP.

### Fluorescence cytometric analysis of Lewis antigens

The cells ($1 \times 10^6$) detached by 2 mmol $l^{-1}$ ethylene diamine tetra-acetic acid (EDTA) were washed and re-suspended in phosphate-buffered saline (PBS) containing 1% BSA and incubated with different monoclonal antibodies of Lewis antigens for 30 min at 4˚C. After 2 washings, the cells were incubated for 45 min at 4˚C with FITC-conjugated goat antibody against mouse IgM (for anti-$Le^x$, anti-$SLe^x$ and anti-$SDLe^x$) or IgG (for anti-$SLe^a$). Then the cells were subjected to flow-cytometry for fluorescence cytometric analysis after suitable washing. Data were expressed as the relative fluorescence intensity.

### Preparation of $^{32}$P-labelled probes of α-1,3/1,4 FuTs

The plasmid containing cDNA of α1,3 FucT-VII (pUC19/FucT-VII) or α1,3/1,4 FucT-III (pUC19/FucT-III) was cut with *Bam*H1 and *Eco*RI, and the cDNA was purified with agarose electrophoretic separation, Tris buffer saturated phenol/chloroform extraction and ethanol precipitation. The probes were labelled with (α-$^{32}$P)-dATP using random primer labelling kit from Promega according to the instructions described in the manual. The cDNA of GAPDH was labelled with the same method.

### RNA extraction and Northern blot of α-1,3/1,4 FuT mRNAs

Total RNA was extracted from the cells using Trizol according to the protocol provided by Promega. Northern blot analysis was carried out with the method described by Sagerstrom and Sive (1996, pp 83–103). Briefly, total RNA (30–50 μg) was separated by formaldehyde denatured electrophoresis, then transferred to Hybond-N$^+$ nylon membrane, and pre-hybridized for 4–6 h at 65˚C in 0.2 mol $l^{-1}$ pH 7.4 sodium phosphate buffer/1 mM EDTA/1% BSA/7% SDS/15% formamide. Hybridization was performed at 65˚C for 16–20 h in the same hybridization solution containing adequate (α-$^{32}$P) labelled probe. After exposure of the membrane under X-ray film, the intensity of each hybridized spot was quantified using a densitometer, and expressed as the relative absorbance unit (absorbance of α FucT/absorbance of GAPDH). The ratio of the untreated control was set as 100%.

The probe used for detection of αFuT-III, V and VI was the α1,3/1,4FuT-III cDNA, and the probes for measurement of α1,3 FuT-IV and α1,3 FuT-VII were the cDNA of αFuT-IV and αFuT-VII respectively. The GAPDH mRNA was determined with GAPDH cDNA for intrinsic control.

### Determination of cell adhesion to HUVEC

Assay of cell adhesion to HUVEC was carried out according to the method reported by Takada et al (1993) with minor modification (Liu et al, 2000). Chiefly, HUVEC were coated onto 96-well plates and treated with 200 ng $ml^{-1}$ TNF-α for 4 h to stimulate the expression of E-selectin on the cell surface. Then 100 μl of the cultured 7721 cells ($1 \times 10^5$ well$^{-1}$) were added to the plate wells and further incubated for 30 min at 4˚C (to minimize possible adhesion mediated by integrins). After washing with PBS 5 times,

© 2001 Cancer Research Campaign 

the cells were fixed with glutaraldehyde and stained with crystal violet. The numbers of cells adhered to HUVEC were counted in 8 high-powered fields (HPFs) (×200). The data were expressed by the mean value of cells per HPF in triplicate with 2 independent experiments.

In order to determine the inhibition of cell adhesion by the different monoclonal antibodies of Lewis antigens and the role of sialyl residue in the adhesion, 7721 cells were pre-incubated with 10 μg $ml^{-1}$ different antibodies for 30 min at 4˚C or with 1 U $ml^{-1}$ sialidase for 1 h at 37˚C before added to the coated HUVEC.

### Measurement of cell migration and invasion

The chemotaxic cell migration assay was performed using 24-well transwell units with polycarbonate filter of 8 μm pore size according to the method of Yu et al (1994) and described in our previous paper (Liu et al, 2000). Each lower compartment of the transwell contained 600 μl of 0.5% FCS as chemoattractant, or 0.5% BSA as negative control in DMEM. Cells ($2 \times 10^4$) in 0.1 ml DMEM–0.1% BSA were added into the upper compartment of the transwell unit and incubated for 6 h at 37˚C in a humidified atmosphere containing 5% $CO_2$. The cells were then fixed with glutaraldehyde and stained with crystal violet. Then the numbers of the cells that had migrated to the lower side of the polycarbonate filter were counted in 8 HPFs (×200). The data were expressed by the mean value of cells per HPF in triplicate with 2 independent experiments.

The procedure for chemotaxic cell invasion text was the same as in the chemotaxic cell migration assay, except that the upper side of polycarbonate filter was coated with 0.1 ml (20 μg $filter^{-1}$) of matrigel in cold DMEM to form a continuous thin layer (Yu et al, 1994). The added cells were $1 \times 10^5$ in 0.1 ml, the FCS used was 1%, and the incubation time was prolonged to 36 h. Cells were stained and counted as described above, and the number of cells invading the lower side of the filter was a measure of the invasive activity of the cells.

### RESULTS

### Expressions of surface Lewis antigens before and after treatment with ATRA or EGF

Using different monoclonal antibodies, CD15 (anti-$Le^x$), KM93 (anti-$SLe^x$), FH6 (anti-$SDLe^x$) and CA19-9 (anti-$SLe^x$), the expressions of 4 Lewis antigens on the surface of untreated (control) 7721 cells were detected with fluorescence cytometric analysis by means of flow cytometry. It was found that only $SLe^x$ was expressed in a significant amount. The expression of $SDLe^x$ was lower, and the other 2 Lewis antigens, $Le^x$ and $SLe^a$, were only expressed in trace amounts. After treatment with 10 μmol $l^{-1}$ ATRA, the expressions of both $SLe^x$ and $SDLe^x$ were obviously decreased ($P < 0.01$), while these 2 Lewis antigens were increased by the treatment of 10 ng $ml^{-1}$ EGF ($P < 0.05$) (Figure 1, Table 1). The changes in the expression of $Le^x$ and $SLe^a$, however, were not detectable. Therefore, $SLe^x$ was selected as the index to compare the effects of 8-Br-cAMP and PMA.

### Expression of surface $SLe^x$ before and after treatment with 8-Br-cAMP or PMA

As shown in Figure 2A and B, the expression of $SLe^x$ on 7721 cells was decreased to 65.6% of the untreated control cells by the treatment of 0.5 mmol $l^{-1}$ of 8-Br-cAMP ($P < 0.05$), but increased to 471.7% of the control value after treated with 100 nmol $l^{-1}$ PMA ($P < 0.01$).

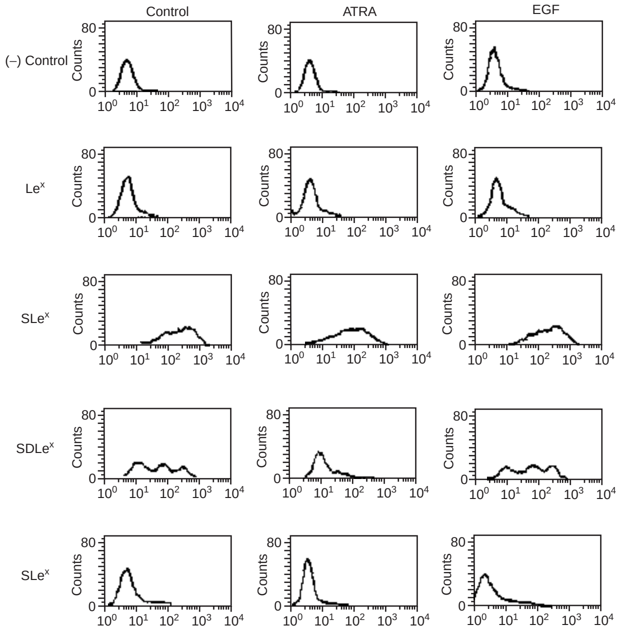

**Figure 1** Expression of 4 Lewis antigens on the surface of 7721 cells before and after the treatment of ATRA or EGF (profiles of flow-cytometric analysis). (–) Control: without the addition of the first antibody; Control: untreated cells; ATRA: 10 μmol $l^{-1}$ ATRA treated cells; EGF: 10 ng $ml^{-1}$ EGF treated cells. The duration of ATRA or EGF treatment was 48 h. The monoclonal antibodies for the detection of $Le^x$, $SLe^x$, $SDLe^x$ and $SLe^a$ were CD15, KM93, FH6 and CA19-9 respectively. The profiles are the representatives of 3 independent experiments. The experimental procedure is described in 'Materials and Methods'

**Table 1** Expressions of $Le^x$, $SLe^x$, $SDLe^x$ and $SLe^a$ in 7721 cells before and after treatment with ATRA or EGF

| Lewis antigen | Control | ATRA (10 μmol$^{-1}$) | EGF (10 ng ml$^{-1}$) |
|---|---|---|---|
| $Le^x$ | Trace | Trace | Trace |
| $SLe^x$ | 241.6 ± 28.3 (100) | 153.9 ± 16.1 (63.7)** | 327.5 ± 35.0 (135.6)* |
| $SDLe^x$ | 76.4 ± 9.5 (100) | 29.7 ± 3.6 (38.8)** | 102.1 ± 13.3 (133.6)* |
| $SLe^a$ | Trace | Trace | Trace |

Data in the table are the mean ± SD of the fluorescence intensity in 3 independent experiments. Data in parentheses are the percentage of control. *: $P < 0.05$, **: $P < 0.01$ as compared with the control.

### Expression of α1,3 FuT-VII mRNA after treatment with ATRA, EGF, 8-Br-cAMP and PMA

$SLe^x$ can be synthesized by αFuT-III, V, VI and VII (Weston et al, 1992a, 1992b; Sasaki et al, 1994; Narimatsu, 1998). In order to study the enzymatic mechanism of the altered expression of $SLe^x$, the expression of αFuT mRNAs were determined using Northern blot, followed by hybridization with different cDNAs of α-1,3/1,4 FuT as probes. It was reported that there was cross-hybridization among the c-DNA of αFuT-III, V and VI owing to the very high (85–90%) sequence homology among these 3 αFuT subtypes (Weston 1992a, 1992b; Narimatsu, 1998), but they can be

 *© 2001 Cancer Research Campaign*

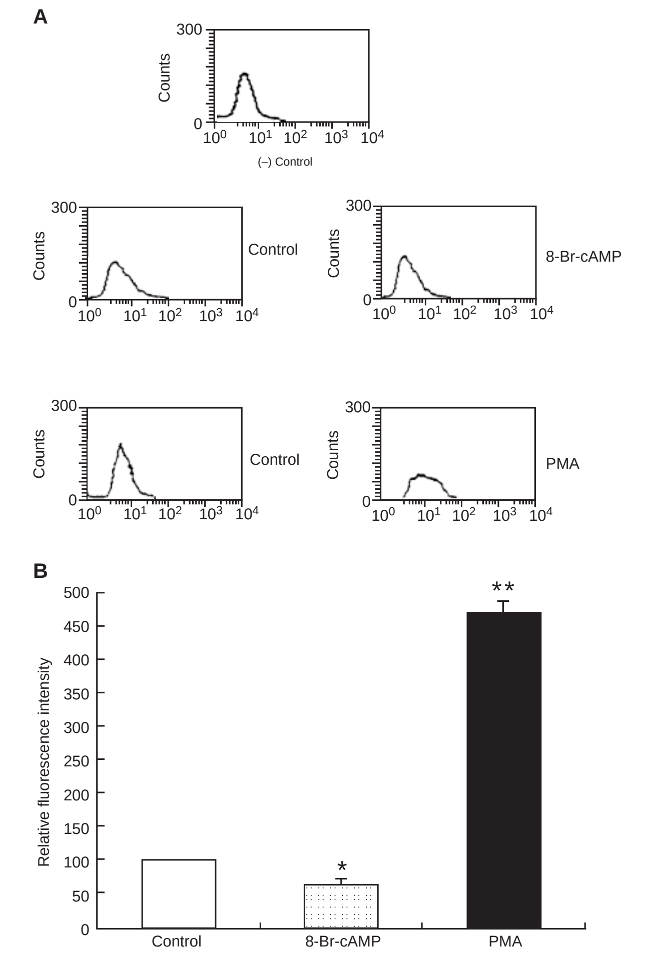

**Figure 2** Expression of SLe$^x$ on the surface of 7721 cells before and after the treatment 8-Br-cAMP and PMA. (**A**) Profiles of flow-cytometric analysis. (–) Control: without the addition of the first antibody; Control: untreated cells; 8-Br-cAMP: 0.5 mmol l$^{-1}$ 8-Br-cAMP treated cells; PMA: 100 nmol l$^{-1}$ PMA-treated cells. The duration of 8-Br-cAMP and PMA treatment was 48 h. The monoclonal antibodies used for detection of SLe$^x$, was FM93. The profiles are the representatives of 3 independent experiments. (**B**) Calculation of relative fluorescence intensity. The mean fluorescence intensity of the control was set at 100%. *: $P < 0.05$, **: $P < 0.01$ as compared with the control. The experimental procedure is described in 'Materials and Methods'

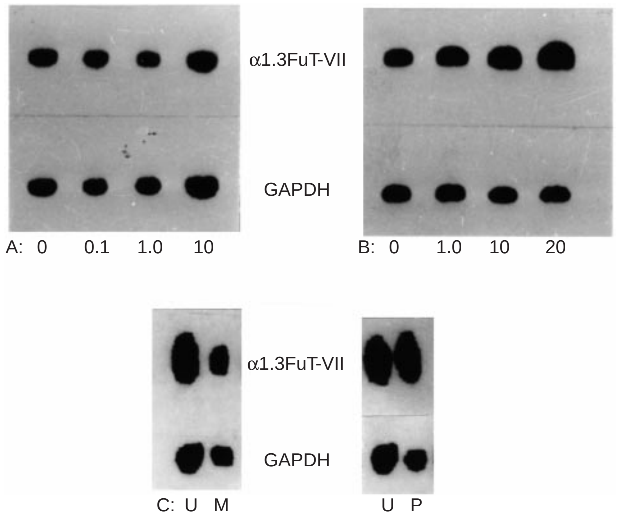

**Figure 3** Expression of α1,3-FucT-VII mRNA in ATRA, EGF, 8-Br-cAMP and PMA treated 7721 cells. (**A**) ATRA-treated cells. The numbers under the spots are the concentrations of ATRA in μmol l$^{-1}$. (**B**) EGF-treated cells. The numbers under the spots are the concentrations of EGF in ng ml$^{-1}$. (**C**) U: untreated cells; M: 0.5 mmol l$^{-1}$ 8-Br-cAMP-treated cells; P: 100 nmol l$^{-1}$ PMA-treated cells. GAPDH: mRNA of glyceraldehyde-3-phosphate dehydrogenase. The duration of inducer treatment was 48 h. The figure only shows one of the 3 repeatable experiments. The experimental procedure is described in 'Materials and Methods'. The probe used was the cDNA of α1,3-FucT-VII

**Table 2** Relative expressions of α1,3 FuT-VII mRNA in 7721 cells after the treatment of differentiation- or proliferation-inducers

| Cell treatment | Relative expression[a] % of control |
|---|---|
| Control | 100.0 |
| Differentiation-inducer | |
| ATRA (0.1 μM) | 84.9 ± 9.1 |
| ATRA (1.0 μM) | 71.3 ± 8.5* |
| ATRA (10.0 μM) | 64.5 ± 7.3** |
| 8-Br-cAMP (0.5 mM) | 74.7 ± 8.2* |
| Proliferation-inducer | |
| EGF (1.0 ng ml$^{-1}$) | 105.7 ± 12.3 |
| EGF (10 ng ml$^{-1}$) | 134.4 ± 14.8* |
| EGF (20 ng ml$^{-1}$) | 169.1 ± 15.2** |
| PMA (100 nM) | 271.9 ± 33.3** |

[a]Calculated as the ratio of absorbance units of α1,3FuT-VII mRNA spot/absorbance units of GAPDH mRNA spot, and set the ratio of the control cells as 100%. The data are the mean ± SD of 3 independent experiments. *: $P < 0.05$, **: $P < 0.01$ as compared with the control.

differentiated with competitive reverse transcription polymerase chain reaction (Narimatsu, 1998). By using this method, it was found that the α1,3 FuT-V gene was not expressed in many tissues, and might be a silent gene (Narimatsu, 1998). Therefore, the amount of mRNA hybridized with α1,3/1,4FuT-III cDNA was mainly the sum of α1,3/1,4FuT-III and α1,3FuT-VI mRNA. However, α1,3 FuT-VII only shares 42–43% and 47% identity in amino acid sequence with αFuT-III/V/VI and α1,3 FuT-IV respectively (Sasaki et al, 1994), its probe is specific, and does not cross-hybrid with other α FuTs.

We found that there was a medium expression of α1,3FuT-VII mRNA in untreated control 7721 cells, which was down-regulated after the treatment of ATRA in a dose-dependent manner (Figure 3A), and up-regulated in the EGF-treated cells with a similar dose-dependent manner (Figure 3B). The reduced and enhanced expressions of α1,3 FuT-VII mRNA were also observed after 8-Br-cAMP and PMA treatment respectively (Figure 3C). The relative expressions of α1,3 FuT-VII mRNA in 7721 cells treated with the above 4 inducers were summarized in Table 2. These results were compatible with the findings that the expressions of SLe$^x$ were lower in ATRA or 8-Br-cAMP treated cells and higher in EGF- or PMA-treated cells.

### Expression of α-FuT-III/VI mRNAs after treatment with ATRA, EGF, 8-Br-AMP and PMA

As shown in Figure 4, the expression of αFuT-III/VI mRNA was rather low in 7721 cells as compared with α1,3 FuT-VII. Its expression was also suppressed by ATRA (Figure 4A) and 8-Br-cAMP (Figure 4B), being 63.2% and 66.7% respectively of the untreated control cells (Table 3), while it was promoted by EGF

*© 2001 Cancer Research Campaign*

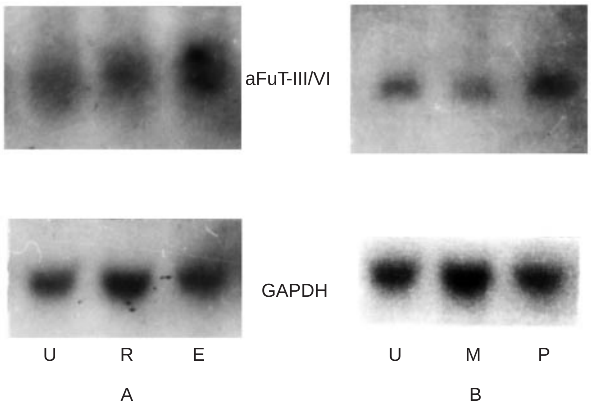

**Figure 4** Expression of α-FucT-III/VI mRNA in ATRA, EGF, 8-Br-cAMP- and PMA-treated 7721 cells. (**A**) after ATRA or EGF treatment. U: untreated cells; R: 10 μmol $l^{-1}$ ATRA treated cells; E: 10 ng $ml^{-1}$ EGF-treated cells. (**B**) after 8-Br-cAMP and PMA treatment. U: untreated cells; M: 0.5 mmol $l^{-1}$ 8-Br-cAMP-treated cells; P: 100 nmol $l^{-1}$ PMA-treated cells. GAPDH: mRNA of glyceraldehyde-3-phosphate dehydrogenase. The duration of inducer treatment was 48 h. The figure only shows one of the 3 repeatable experiments. The experimental procedure is described under 'Materials and Methods'. The probe used was the cDNA of α1,3/1,4-FucT-III

**Table 3** Relative expressions of α-FuT-III/VI mRNA in 7721 cells after the treatment of differentiation- or proliferation-inducers

| Cell treatment | Relative expression[a] % of control |
|---|---|
| Control | 100.0 |
| Differentiation-inducer | |
| ATRA (10 μM) | 63.2 ± 7.1* |
| 8-Br-cAMP (0.5 mM) | 66.7 ± 7.8* |
| Proliferation-inducer | |
| EGF (10 ng $ml^{-1}$) | 151.5 ± 14.2* |
| PMA (100 nM) | 177.4 ± 19.0** |

[a]Calculated as the ratio of absorbance units of αFuT-III/VI mRNA spot/absorbance units of GAPDH mRNA spot, and set the ratio of the control cells as 100%. The data are the mean ± SD of 3 independent experiments. *: $P < 0.05$, **: $P < 0.01$ as compared with the control.

(Figure 4A) and PMA (Figure 4B), being 151.5% and 177.4% respectively of the untreated control value (Table 3).

The transcript of α1,3 FuT-IV was not detected in control and treated 7721 cells by using α1,3 FuT-IV cDNA as probe (data not shown).

### Alteration of cell adhesion to HUVEC after treatment with ATRA, 8-Br-cAMP, EGF and PMA

To mimic the Lewis antigen/selectin interaction in vivo, tumour necrosis factor-α (TNF-α) was used for stimulating the expression of E-selectin on the surface of human umbilical vein endothelial cells (HUVEC), and determined the attachment of 7721 cells to the coated HUVEC. It was observed that the adhesions of ATRA and 8-Br-cAMP treated 7721 cells to HUVEC were decreased by 48.1% and 45.0% respectively ($P < 0.01$), while those of EGF- and PMA-treated 7721 cells was increased by 71.3 and 86.8% respectively ($P < 0.01$) (Figure 5).

When different monoclonal antibodies of Lewis antigens were added to block the surface Lewis antigens on 7721 cells, it was found that after the HUVEC were stimulated with TNF-α, only

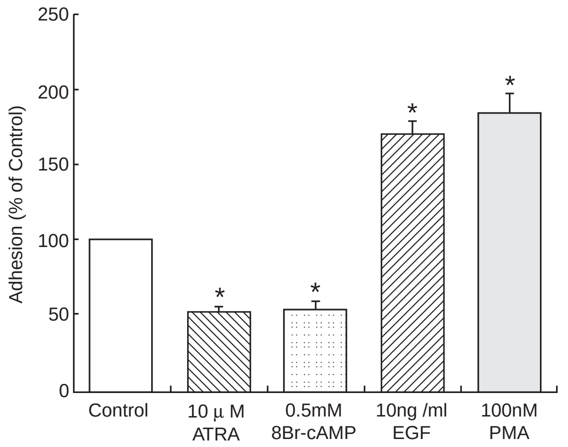

**Figure 5** Adhesion of 7721 cells to HUVEC after treatment with ATRA, 8-Br-cAMP, EGF and PMA. *: $P < 0.01$ compared with control ($n = 6$). The duration of inducer treatment was 48 h. The experimental procedure is described in 'Materials and Methods'

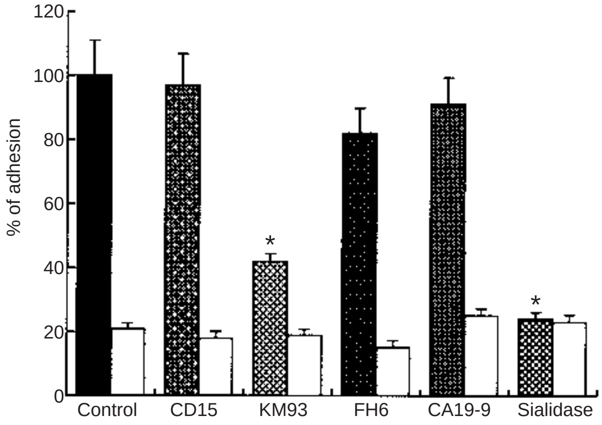

**Figure 6** Effect of monoclonal antibodies and sialidase on the adhesion of 7721 cells to HUVEC. Open bar: without pre-incubation of TNF-α, Filled bar: with pre-incubation of TNF-α. CD15: mAb of $Le^x$, KM93: mAb of $SLe^x$, FH6: mAb of $SDLe^x$, CA-19-9: mAb of $SLe^a$. The final concentration of antibodies was 10 μg $ml^{-1}$. The concentration of sialidase was 1 U $ml^{-1}$. *: $P < 0.01$ compared with control. The experimental procedure is described in 'Materials and Methods'

KM93 (anti-$SLe^x$ mAb) showed a significant inhibition on the adhesion of 7721 cells to HUVEC ($P < 0.01$). FH6 (anti-$SDLe^x$) slightly suppress the adhesion but with no statistical significance ($P > 0.05$). In contrast, other antibodies (anti-$Le^x$, CD15 and anti-$SLe^a$, CA19–9) did not show obvious blocking effects on the cell adhesion. If the experiment was carried out in the absence of TNF-α, or in the presence of sialidase to remove the terminal sialyl residues on Lewis antigens, only a little cell adhesion was observed and showed no difference among the samples added different mAbs (Figure 6).

### Alteration of cell migration and invasion after treatment with ATRA, 8-Br-cAMP, EGF, and PMA

As indicated in Figure 7, the ability of cell migration through transwell was declined by the treatment of ATRA and 8-Br-cAMP (51.7% and 62.1% of the control value respectively, $P < 0.05$), but elevated by EGF and PMA treatment (193.1% and 186.2% of the

*© 2001 Cancer Research Campaign*

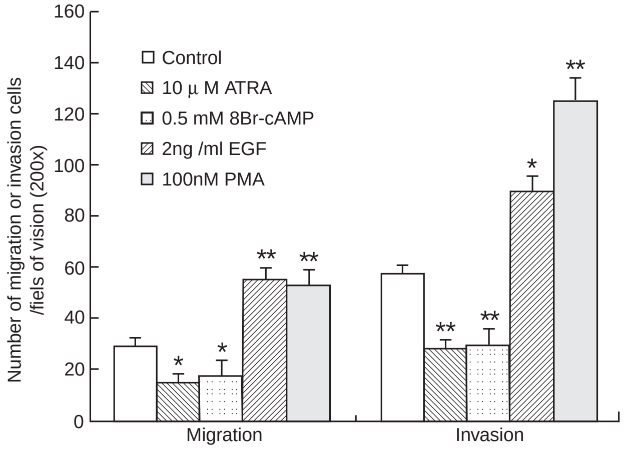

**Figure 7** Migration and invasion of 7721 cells after treatment with ATRA, 8-Br-cAMP, EGF and PMA. *: $P < 0.05$ compared with control; **: $P < 0.01$ compared with control ($n = 6$). The duration of inducer treatment was 48 h. The experimental procedure is described in 'Materials and Methods'

control, $P < 0.01$). Similar to cell migration, cell invasion through matrigel membrane was also reduced after the treatment of ATRA and 8-Br-cAMP (50.0% and 53.4% of the control respectively, $P < 0.01$), but enhanced by the treatment of EGF and PMA (156.9% and 219.0%, $P < 0.05$ and $P < 0.01$ respectively).

### DISCUSSION

Our findings revealed that 7721 cells express mainly $SLe^x$ on the surface, and a small amount of $SDLe^x$, but the expression of $Le^x$ and $SLe^a$ were very low or only trace amount. The expression of Lewis antigen, $SLe^x$, on 7721 cell surface was down-regulated by the treatment of differentiation-inducers, ATRA and 8-Br-cAMP, while up-regulated by the treatment of proliferation-inducers, EGF and PMA. These compounds also regulated the expression of $SDLe^x$ in the same direction, but to a lesser extent.

The main enzymatic mechanism of the alteration of $SLe^x$ expression was demonstrated to be the change in α1,3 FuT-VII expression, because α1,3 FuT-VII was obviously expressed in 7721 cells, in contrast to the low expression of α1,3 FuT-III/VI. The substrate specificity of α1,3 FuT-VII is very high, and $SLe^x$ is the only Lewis antigen synthesized by α1,3 FuT-VII (Sasaki et al, 1994). Furthermore, α1,3 FuT-IV as well as α1,3 FuT-IX, in general, can not synthesize any sialyl Lewis antigens (Narimatsu, 1998). However, αFuT-III/VI might have a minor contribution to the regulation of $SLe^x$ in 7721 cells, because ATRA, 8Br-cAMP down-regulate, and EGF, PMA up-regulate this enzyme, too. αFuT-III/VI is also considered to be responsible for the expression change in $SDLe^x$, since it was reported that α1,3 FuT-VI is the major enzyme for the synthesis of $SDLe^x$ from $SLe^x$ (Weston et al, 1992b). Therefore, our results of measuring the mRNAs of αFuT subtypes were consistent with the observations of Lewis antigen expressions on 7721 cell surface. After the treatment of 10 μmol $l^{-1}$ ATRA or 10 ng $ml^{-1}$ EGF, the percentage of decrease or increase in α1,3 FuT-VII expression was very closed to the percentage change in $SLe^x$ expression, when the data in Table 2 and Table 1 were compared. It was found that that the changes in α1,3 FuT-VII and $SLe^x$ were –35.5% and –36.3% respectively for 10 μmol $l^{-1}$ ATRA, while +34.4% and +35.6% respectively for 10 ng $ml^{-1}$ EGF. However, the changes in α-1,3 FuT-VII and $SLe^x$ expressions in PMA treated cells (+171.9% and +371.7% respectively) were not closed to each other when compared the data in Table 2 and Figure 2B. This discrepancy suggests that probably other enzyme(s) regulated by PMA may participate in the control of $SLe^x$ synthesis, such as α2,3 sialyltransferase.

After the treatment of differentiation-and proliferation-inducers, the altered αFuT-III/VI expression was not compatible with the unchanged $SLe^a$ expression, though $SLe^a$ only can be synthesized by α1,3/1,4 FuT-III. This may be explained by the following reasons. First, the amount of $SLe^a$ on 7721 cell surface was too low for the detection of its decrease, and even its increase. Second, the rate-limiting enzyme responsible for $SLe^a$ synthesis is not α1,3/1,4 FuT-III, but β1,3-galatosyltransferase 5, which synthesizes the precursor of $SLe^a$, type I sugar chain, GalβI, 3GlcNAc (different from the type II chain, Galβ1, 4GlcNAc, in $Le^x$ and $SLe^x$). The expression of β1,3-galatosyltransferase 5 is in accordance with the expression of $SLe^a$ in many cell lines (Issiki et al, 1999).

The mechanism of the change in cell adhesion to HUVEC by the above mentioned 4 inducers is considered to result from the altered synthesis of $SLe^x$, since the adhesion of 7721 cells to HUVEC was only significantly abolished by the antibody of $SLe^x$, KM93, but not by other antibodies of Lewis antigens. It indicates that $SLe^x$ is the most important Lewis antigen responsible for the adhesion of 7721 cell to HUVEC among the 4 antigens assayed in our laboratory. α1,3 FuT-VII not only contributes greatly to the synthesis of $SLe^x$, but also anticipates in the regulation of metastasis-related phenotypes, which are also assumed to be mediated by $SLe^x$. This is evidenced by our findings that the increase of $SLe^x$ expression was accompanied with the enhanced cell adhesion to HUVEC, as well as cell migration and invasion after transfection of α1,3 FuT-VII cDNA into 7721 cells, Moreover, the migration and invasion of 7721 cells were also suppressed by anti-$SLe^x$, KM93, but not by the other monoclonal antibodies (to be published).

The abolishment of cell adhesion to HUVEC by sialidase treatment revealed that the sialy residue in Lewis antigens is a critical glycosyl residue in the cell adhesion to HUVEC.

The metastasis potential of transformed cells can be assayed in vivo by inoculation of the modified (reagent-treated or gene-transfected) cells into nude mice or other immuno-permissive animals to observe the number of metastatic foci in the lung. However, the ability of cell adhesion, cell migration and invasion are good indexes for the estimating of metastasis-potential ex vivo and are widely used in a lot of laboratories, and the results of these ex vivo experiments are always in accordance with the in vivo assay (Welch, 1997). The assay of cell adhesion to TNF-α stimulated HUVEC mimics the interaction between the Lewis antigens on malignant cells and the E-selectin on vascular endothelium in vivo. The cell invasion assay is similar to the penetration of cells through the vascular membrane, since matrigel is an artificial membrane resembling the extracellular interstitial membrane coated on the epithelial cells.

ATRA and cAMP derivatives are the differentiation inducers of 7721 cells (Ai et al, 1990, 1991a; Chai et al, 1993a; Chai and Chen, 1994a, 1994b). It is reasonable to consider that these 2 compounds may have an anti-metastasis effect on the same cell line, because metastasis is a behaviour of malignant cells, and differentiation of cancer cells is often accompanied by the decrease of malignancy. Our laboratory has discovered that ATRA up-regulate the expression of a metastasis-suppressive gene, *nm23*-H1, in 7721 cells, and this is supposed to be one of the

*© 2001 Cancer Research Campaign*

mechanisms of the decreased metastasis-related phenotypes after ATRA treatment (Liu et al, 2000). Moreover, the transfection of *nm23*-H1 cDNA into 7721 cells leaded to the similar results as the treatment of ATRA, including the decreased expressions of $SLe^x$ and α1,3 FuT-VII (unpublished data). Oppositely, EGF down-regulates the expression of nm23-H1 in 7721 cells (Liu et al, 2000), and this can explain the increased metastasis-related phenotypes provoked by EGF. The proliferation inducers usually increase the malignancy and metastasis potency, as it was evidenced in this paper. We also found that the expressions of $SLe^x$/α1,3 FuT-VII and above metastasis potential-related phenotypes were increased after the transfection of an oncogene, *c-erb*B2/*neu*, into 7721 cells (to be published).

The molecules for transducing the signals of 8-Br-cAMP and PMA are protein kinase A (PKA) and protein kinase C (PKC) respectively (Nishizuka, 1992; Nicholas and Paolo, 1996). The signal transduction pathway for EGF is receptor tyrosine protein kinase (R-TPK)→Grb2/Sos adaptor proteins→ Ras→Raf MEK→ERK, or R-TPK→ phosphatidylinositol 3-kinase→protein kinase B (PKB or Akt) pathway (van der Geer et al, 1994). ATRA down-regulated PKC (Chai and Chen, 1994a) and R-TPK (Chai et al, 1993a), also PKB (unpublished data), which are probably the mechanisms of the antagonistic effect of ATRA against EGF and PMA in the expression of metastasis-related phenotypes. Therefore, ATRA or its derivatives may become an anti-metastasis drug used clinically in the prevention or treatment of metastasis.

The molecular mechanisms of metastasis are very complex, and the glycan structures on cell surface play an important role in the metastasis-related phenotypes, including cell adhesion, migration and invasion, etc. However, the alteration in the surface Lewis antigens is at least one of their molecular bases in addition to the well-documented GlcNAcβ1,6Manα-branch of N-glycans.

### ACKNOWLEDGEMENTS

This work was supported by the grant from National Natural Science Foundation of China (No. 39630080), Education Committee of Shanghai Municipal Government (No. 139808010) and the ninth 5-years plan of National Science and Technology (96–906–01–15).

### REFERENCES

Ai ZW, Zha XL, Liu Y and Chen HL (1990) Effects of retinoic acid on hepatocarcinoma cell line. *J Tumor Mark Oncol* **5**: 59–70

Ai ZW, Zha XL, Tang H and Chen HL (1991a) Reversing effect of retinoic acid on some phenotypes of human hepatocarcinoma cell line. *Chin J Oncol* **13**: 9–11

Ai ZW, Chen HL, Gu JR, Jiang HO and Long J (1991b) Inhibition by retinoic acid of the expression of the oncogene ras in human hepatocarcinoma cell line. *Acta Biochim Biophy Sinica* **23**: 453–457

Chai XY, Qin H and Chen HL (1993a) Protein kinase and cell differentiation, inhibition of tyrosine protein kinase by two inducers of differentiation. *Chin Biochem J* **9**: 162–167

Chai XY, Shen YF and Chen HL (1993b) Effect of phorbor ester on protein kinase C and tyrosine protein kinase in human hepatocarcinoma cell line. *Chin J Oncol* **15**: 182–184

Chai XY and Chen HL (1994a) The effects of two differentiation-inducers on serine-protein kinase activity in human hepatocarcinoma cell line. *Acta Acad Med Shanghai* **21**: 170–175

Chai XY and Chen HL (1994b) Expression of oncogenes during induced differentiation of human hepatocarcinoma cell line. *Chin J Cancer Res* **6**: 3–8

Chai XY, Chen HL, Zhou XM, Qian LF, Chen SH, Jiang HO and Gu JR (1994c) The opposite effects of retinoic acid and phorbol ester on the expression of c-myc and IGF-II genes in human hepatocarcinoma cells. *Acta Oncol Sinica* **4**: 229–231

Chen HL, Dong SC, Ju TZ and Yang XP (1995) Effect of retinoic acid on the structure of N-glycan on the surface of human hepatocarcinoma cells and its enzymatic Mechanism. *J Cancer Res Clin Oncol* **121**: 397–401

Dong SC, Yang XP and Chen HL (1994) Effect of dibutyryl cAMP on the type and antennary number of N-glycans on the surface of human hepatocarcinoma cell line SMMC 7721. *Acta Biochim Biophys Sinica* **26**: 43–49

Hakomori SI (1996) Tumor malignancy defined by aberrant glycosylation and sphingo(glyco)lipid metabolism. *Cancer Res* **56**: 5309–5318

Issiki S, Togayachi A, Kudo T, Nishihara S, Watanabe M, Kobata T, Kitajima M, Shiraishi H, Sasaki K, Andoh T and Narimatsu H (1999) Cloning, expression, and charisterazation of novel UDP-galactose: β-N-actylglucosamine β1,3-galactosyltransferase (β3Gal-T5) responsible for synthesis of type I chain in colorectal and pancreatic epithelia and tumor cells derived therefrom. *J Biol Chem* **274**: 12499–12507

Ito H, Hiraiwa N, Sawada-Kasugai M, Akamatsu S, Tachikawa T, Kasai Y, Akiyama S, Ito K, Takagi H and Kannagi R (1997) Altered mRNA expression of specific molecular species of fucosyl-and sialyl-transferases in human colorectal cancer tissues. *Int J Cancer* **71**: 5560–5564

Jorgensen T, Berner A, Kaalhus O, Tveter KJ, Danielsen HE and Bryne M (1995) Up-regulation of the oligosaccharide sialyl Lewis X: a new prognosis parameter in metastatic prostate cancer. *Cancer Res* **55**: 1817–1819

Kansas GS. Selectins and their ligands: (1996) Current concepts and controversies. *Blood* **88**: 3259–3287

Kudo K, Ikehara Y, Togayachi A, Kaneko M, Hiraga T, Sasaki K and Narimatsu H (1998) Expression, cloning and characterization of a novel murine α1,3-fucosyltransferase, mFuc-IX, that synthesis the Lewis X (CD15) epitope in brain and kidney. *J Biol Chem* **273**: 26729–26738

Kudo T, Ikehara Y, Togayachi A, Morozumi K, Watanabe M, Nakamura S and Narimatsu H (1998) Up-regulation of a set of glycosyltransferase gene in human colorectal cancer. *Lab Invest* **78**: 797–811

Kukowska-Latallo JF, Larsen RD, Nair RP and Lowe JB (1990) A cloned human cDNA determines expression of a mouse stage-specific embryonic antigen and the Lewis blood group (α1,3/1,4) fucosyltransferase. *Genes Develop* **4**: 1288–1303

Liu F, Qi HL and Chen HL (2000) Effects of all-*trans* retinoic acid and epidermal growth factor on the expression of *nm23*-H1 in human hepatocarcinoma cells. *J Cancer Res Clin Oncol* **126**: 85–90

Lowe JB, Kukowska-Latallo JF, Nair RP, Larsen RD, Marks RM, Marcher BA, Kelly RJ and Ernst LK (1991) Molecular cloning of a human fucosyltransferase gene that determines expression of the Lewis X and VIM-2 epitopes but not ELAM-1-dependent cell adhesion. *J Biol Chem* **266**: 17467–17477

Nakamori S, Kameyama M, Imaoka S, Furukawa H, Ishikawa O, Sasaki T, Kabuto T, Iwanaga T, Matsushita Y and Irimura Y (1993) Increased expression of sialyl Lewis X antigen correlates with poor survival in patients with colorectal carcinoma: clinico-pathological and immunohisto-chemical study. *Cancer Res* **53**: 3632–3637

Narimatsu H (1998) Human fucosyltransferases, Tissue distribution of blood group antigens, cancer associated antigens and fucosyltransferase. *Protein Nucleic Acid Enzyme* **48**: 2394–2403

Nicholas SF and Paolo SC (1996) Transcription factors coupled to the cAMP signalling pathway. *Biochim Biophys Acta* **1288**: F101–F121

Nishizuka Y (1992) Intracellular signaling by hydrolysis of phospholipids and activation of protein kinase C. *Science* **258**: 607–614

Ogawa J, Tsurumi T, Yamada S, Koide S and Shohtsu A (1994) Blood vessel invasion and expression of sialyl Lewis X and proliferating cell nuclear antigen in non-small cell lung cancer. *Cancer* **73**: 1177–1183

Ogawa J, Inoue H and Koide S (1997) α-2,3-sialyltransferase type 3N and α-1,3-fucosyl-transferase type VII are related to sialyl Lewis X synthesis and patient survival from lung carcinoma. *Cancer* **79**: 1678–1685

Sagestrom CG and Sive HL (1996) RNA blot analysis, In *A Laboratory Guide to RNA: Isolation, Analysis, and Synthesis*. Krieg PA (ed) pp 83–103. Wiley-Liss Inc. London, New York, Tokyo

Sasaki K, Kurata K, Funayama K, Nagata M, Watanabe E, Ohta S, Hanat N and Nishi T (1994) Expression cloning of a novel α1,3-fucosyltransferase that is involved in biosynthesis of the sialyl Lewis X carbohydrate determinants in leukocytes. *J Biol Chem* **269**: 14730–14737

Shen YF, Chai XY and Chen HL (1993) Effect of phorbol ester on tyrosine protein kinase in human hepatocarcinoma cell line. *Acta Biochim Biophy Sinica* **25**: 537–540

Takada A, Ohmori K, Yoneda T, Tsuyuoka K, Hasegawa K, Kiso M and Kannagi R (1993) Contribution of carbohydrate antigens sialyl Lewis A and sialyl Lewis X to adhesion of human cancer cells to vascular endothelium. *Cancer Res* **53**: 354–361

 © 2001 Cancer Research Campaign

Taniguchi N, Miyoshi E, Ko JH, Ikeda Y and Ihara Y (1999) Implication of N-acetylglucosaminyl transferases III and V in cancer: gene regulation and signaling mechanism. *Biochim Biophys Acta* **1455**: 287–300

van der Geer P, Hunter T and Lindberg RA (1994) Receptor protein-tyrosine kinase and their signal transduction pathways. *Ann Rev Cell Biol* **10**: 251–337

Welch DR (1997) Technical considerations for studying cancer metastasis *in vivo*. *Clin Exp Metastasis* **15**: 272–306

Weston BW, Nair RP, Jarsen RD and Lowe JB (1992a) Isolation of a novel human α1,3 fucosyl-transferase gene and molecular comparison to the human Lewis blood group α(1,3/1,4) fucosyltransferase gene, synthetic, homologous, nonallelic genes encoding enzymes with distinct acceptor substrate specificity. *J Biol Chem* **267**: 4152–4160

Weston BW, Smith PL, Kelly RJ and Lowe JB (1992b) Molecular cloning of a fourth member of a human α(1,3) fucosyltransferase gene family. Multiple homologous sequences that determine expression of the Lewis X, sialyl Lewis X, and difucosyl sialyl Lewis X antigens. *J Biol Chem* **267**: 24575–24584

Xia BY, Guo HB and Chen HL (1998) Dual regulation of tyrosine protein kinase and protein kinase C on N-acetylglucosaminyltransferase V. *Acta Biochim Biophs Sinica* **30**: 427–431

Yu D, Wang SS, Dulki KM, Tsai CM, Nicolson GL and Hung MC (1994) C-erbB2/neu overexpression enhances metastatic potencial of human lung cancer cells by induction of metastasis-associated properties. *Cancer Res* **54**: 3260–3266

*© 2001 Cancer Research Campaign* 
